# Supplementary material for: Putting measurement-based care into action: a multi-method study of the benefits of integrating routine client feedback in coordinated specialty care programs for early psychosis
Source: BMC Psychiatry. 2024 Dec 2;24:871. doi: 10.1186/s12888-024-06258-1 (PMC11610165; doi:10.1186/s12888-024-06258-1)
Supplement: Supplementary file 1 — Additional file 1: Supplementary Table 1. Summary of participant samples used in quantitative and qualitative analyses. [file 12888_2024_6258_MOESM1_ESM.docx]

**Additional File 1.**

**Title:** Supplementary Table 1

**Description:** Summary of participant samples used in quantitative and qualitative analyses

| Total number of clients included in the quantitative analyses | 204 |
| --- | --- |
| Number of clients who had data collected before implementation of the feedback report | 133 |
| Dates: January 2018 - November 2021 |  |
| Number of clients who had data collected after implementation of the feedback report | 71 |
| Dates: December 2021 - December 2023 |  |
| Number of clients who did not receive a feedback report | 146 |
| Number of clients who did receive a feedback report | 58 |
| Number of clients who completed self-report measures at intake and after 6 months of treatment | 67 |
| Number of clients whose functioning was rated by clinicians at intake and after 6 months of treatment | 106 |
| Number of clients who completed qualitative interviews | 5 |
| Number of clinicians who completed qualitative interviews | 5 |
| Number of clients who completed the anonymous feedback report survey | 9 |
| Number of clinicians who completed the anonymous feedback report survey | 13 |
