# Supplementary material for: Putting measurement-based care into action: a multi-method study of the benefits of integrating routine client feedback in coordinated specialty care programs for early psychosis
Source: BMC Psychiatry. 2024 Dec 2;24:871. doi: 10.1186/s12888-024-06258-1 (PMC11610165; doi:10.1186/s12888-024-06258-1)
Supplement: Supplementary file 2 — Additional file 2: Supplementary Table 2. Goals and Features of Personalized Feedback Report. [file 12888_2024_6258_MOESM2_ESM.docx]

**Additional File 2.**

**Title:** Supplementary Table 2

**Description:** Goals and Features of Personalized Feedback Report

| **Goals** | - To share findings with clients about current strengths and struggles from MBC assessment and build motivation for treatment. - To identify possible treatment targets with a clinician. - To identify coping strategies for clients to use to address current identified concerns. |
| --- | --- |
| **Anonymous Feedback Survey** | A QR code is included on the first page of the report to elicit feedback from clients, clinicians, and family members to continuously improve the implementation of the personalized feedback report. |
| **Definition of Measure** | Each assessment measure has a brief description to help the client understand the purpose of the measure and how to interpret the scoring. |
| **Data Visualization of Client Scores** | Each assessment includes a data visualization of either a bar graph or normal curve that is color coded based on the client’s score in comparison to averages. Green indicates above average. Blue indicates average. Orange indicates area for improvement. |
| **Recommendations for Treatment** | Each assessment measure includes recommendations based on the client’s score.  The recommendations include simple changes or coping skills or identify a particular treatment module in IRT or referral to connect with Supported Employment or Education. |
| **Summary of Assessment Data** | At the end of the report, there is a table with all of the client’s raw scores and z scores. |
| **Access to Assessment Measures** | The last page of the report lists all the measures with a hyperlink to a pdf of the full measure that includes the questions and answer choices. |
