# Supplementary material for: Putting measurement-based care into action: a multi-method study of the benefits of integrating routine client feedback in coordinated specialty care programs for early psychosis
Source: BMC Psychiatry. 2024 Dec 2;24:871. doi: 10.1186/s12888-024-06258-1 (PMC11610165; doi:10.1186/s12888-024-06258-1)

**Additional File 3.**

**Title:** Personalized Feedback Report example: Motivation

**Description:** Page from a sample feedback report regarding motivation, scored on the Behavioral Inhibition and Activation Scale (BIS/BAS)


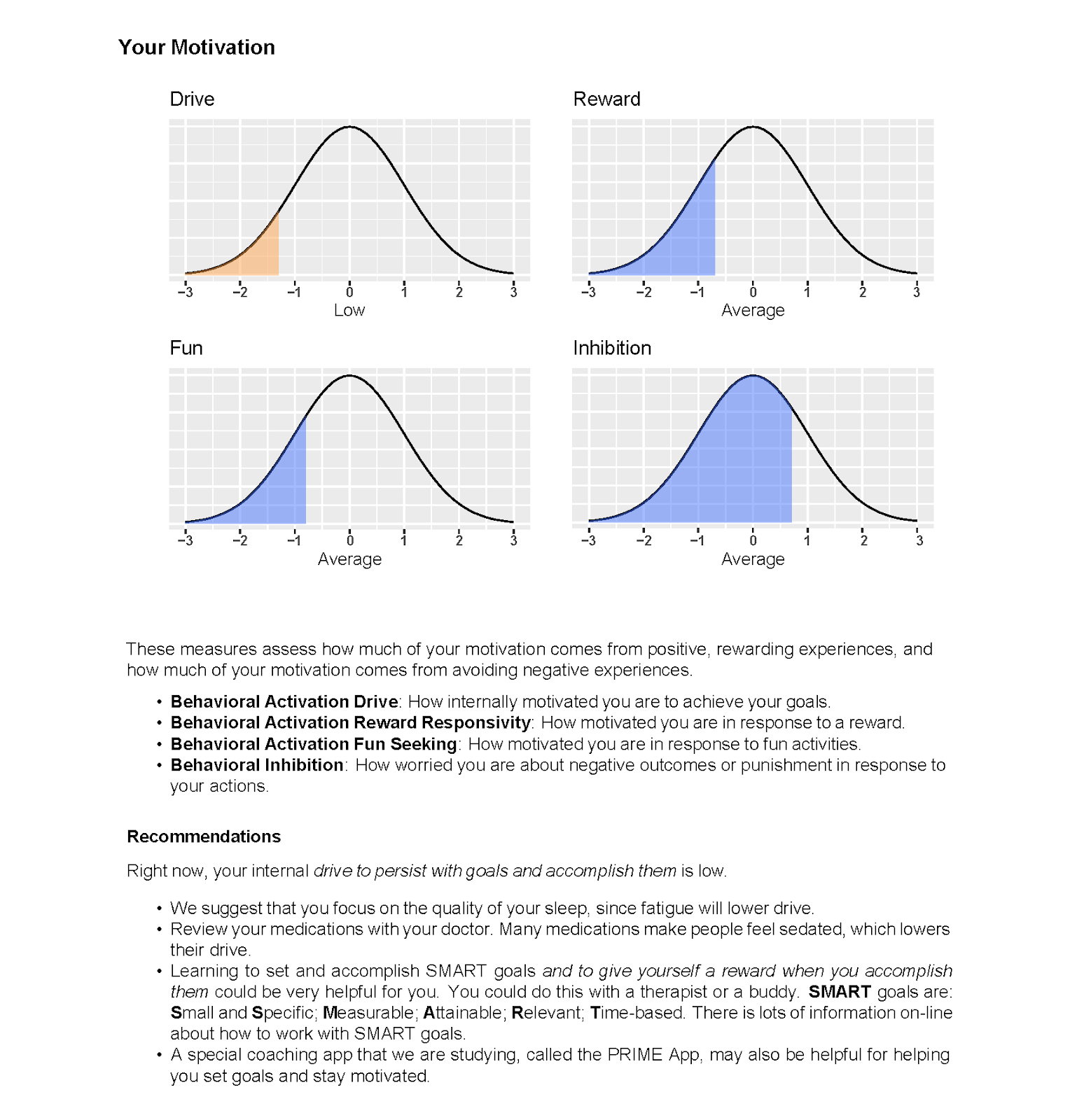

Supplement: Supplementary file 3 — Additional file 3: Personalized feedback report example: Motivation. [file 12888_2024_6258_MOESM3_ESM.docx]
