# Supplementary material for: Putting measurement-based care into action: a multi-method study of the benefits of integrating routine client feedback in coordinated specialty care programs for early psychosis
Source: BMC Psychiatry. 2024 Dec 2;24:871. doi: 10.1186/s12888-024-06258-1 (PMC11610165; doi:10.1186/s12888-024-06258-1)
Supplement: Supplementary file 4 — Additional file 4: Supplementary Table 3. Assessments included in the personalized feedback report. [file 12888_2024_6258_MOESM4_ESM.docx]

**Additional File 4.**

**Title:** Supplementary Table 3

**Description:** Assessments included in the personalized feedback report

| **Feedback Report Measures** |
| --- |
| Minnesota Symptom Severity Scale |
| Brief-COPE |
| Drug Abuse Screening Test (DAST-10) |
| Alcohol Use Disorders Identification Test (Audit-C) |
| PROMIS Sleep Disturbance |
| International physical activity questionnaire (IPAQ) |
| Post Traumatic Stress Disorder Checklist (PCL-5 8 items) OR Child and Adolescent Trauma Screen (CATS) |
| Cognitive measures: processing speed, verbal memory and episodic memory, perceptual reasoning and problem solving, emotion recognition |
| Behavioral Inhibition and Activation Scale (BIS/BAS) |
| Illness Management and Recovery (IMR) - Self Rating |
| Life Event Checklist - LEC |
