# Supplementary material for: Putting measurement-based care into action: a multi-method study of the benefits of integrating routine client feedback in coordinated specialty care programs for early psychosis
Source: BMC Psychiatry. 2024 Dec 2;24:871. doi: 10.1186/s12888-024-06258-1 (PMC11610165; doi:10.1186/s12888-024-06258-1)
Supplement: Supplementary file 5 — Additional file 5. MBC Interview questions. [file 12888_2024_6258_MOESM5_ESM.docx]

**MBC Interview Questions**

*Demographic questions*:

“What is your age?”

“How long have you been participating in the coordinated specialty care program?”

“What is your highest level of education?”

“How would you describe your race?”

*Questions about experience in MBC*:

“How was your experience having your feedback session with your provider/client?”

“Did you notice any change in how connected you felt towards your clinician/client following the feedback session?”

“What was the most meaningful part of the feedback session?”

“What was the least helpful part of the feedback session?”

“What was missing from the feedback session that would be helpful for you in the future?”

*Additional Prompts*:

“Can you tell me more about this experience?”

“Can you provide an example of what you are describing?”
